# First person – Sara Ibrahim

**DOI:** 10.1242/dmm.045195

**Published:** 2020-06-26

## Abstract

First Person is a series of interviews with the first authors of a selection of papers published in Disease Models & Mechanisms, helping early-career researchers promote themselves alongside their papers. Sara Ibrahim is first author on ‘A novel Cre-enabled tetracycline-inducible transgenic system for tissue-specific cytokine expression in the zebrafish: CETI-PIC3’, published in DMM. Sara is an MD-PhD student in the lab of Emily K. Sims and Ryan M. Anderson at Indiana University School of Medicine, Indianapolis, IN, USA, developing and utilizing molecular biology techniques to study β cell dysfunction.


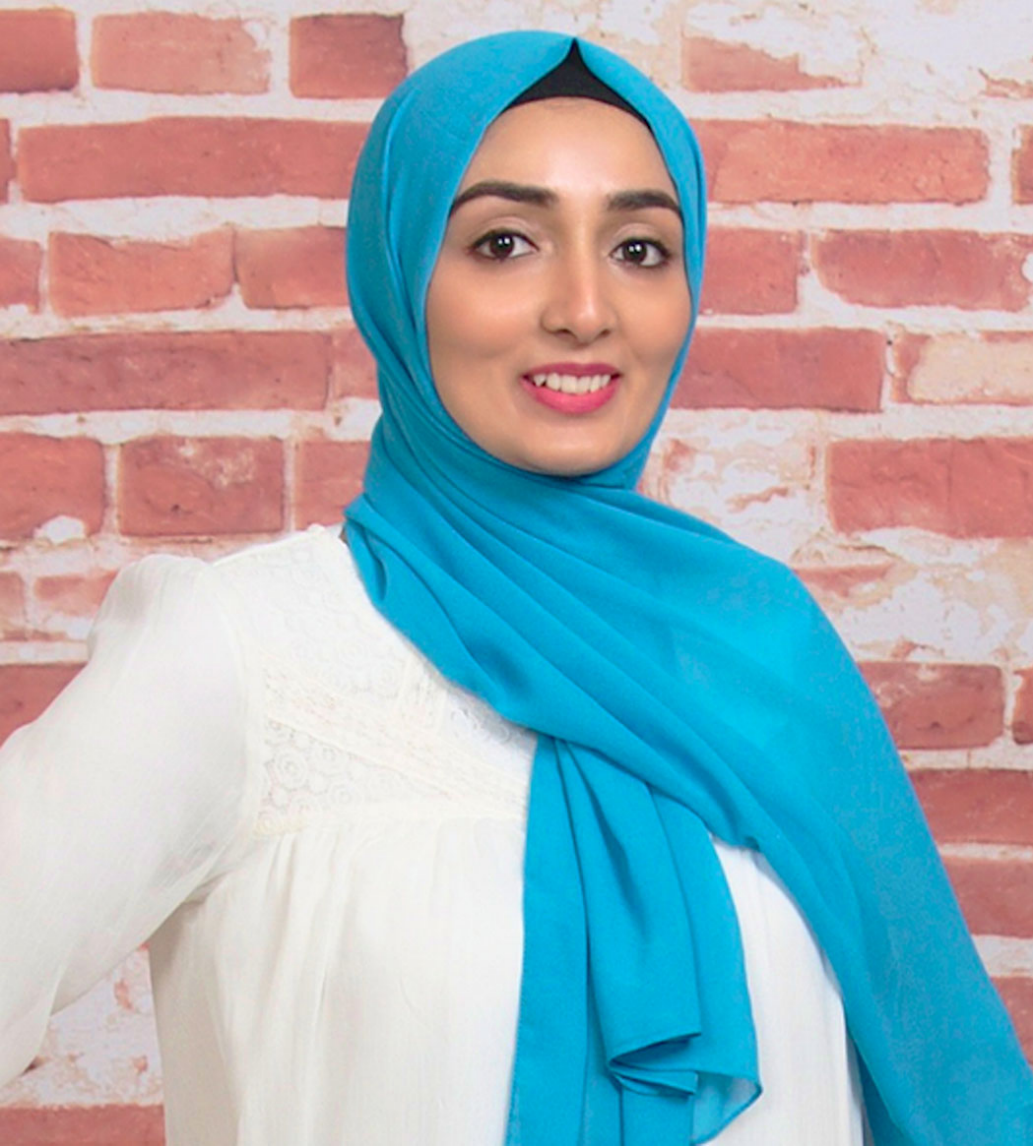


**Sara Ibrahim**

**How would you explain the main findings of your paper to non-scientific family and friends?**

Pro-inflammatory cytokines are signaling molecules that are primarily produced by immune cells (including activated macrophages and T cells) that coordinate and amplify certain immune responses. Dysregulated pro-inflammatory cytokine activity is also involved in several disease processes, such as cell injury, infection, invasion and inflammation. Although pro-inflammatory cytokines are crucial in disease progression for many different pathologies, *in vivo* model systems to study them are lacking. To bridge this gap, in our study, we have developed a zebrafish model system to induce pro-inflammatory cytokines in a titratable and tissue-specific manner.

**What are the potential implications of these results for your field of research?**

Pro-inflammatory cytokines, such as tumor necrosis factor alpha (TNFα), interleukin 1 beta (IL1β) and interferon gamma (IFNɣ), are implicated in the development and progression of multiple inflammatory diseases. However, approaches to induce these cytokines *in vivo* are limited due to the inability to allow for tissue-specific cytokine expression and the inability to precisely titrate the level of induction of these cytokines. Prior to this paper, no models of tissue specific, titratable pro-inflammatory cytokine induction in the zebrafish had previously been reported. In this study, we developed a Cre-enabled and tetracycline-inducible transgenic system for tissue-specific cytokine expression (CETI-PIC3) of *ifng1*, *tnfa* and *il1b*. This model system can be used to study multiple disease states and pathologies with an underlying inflammatory etiology.

**What are the main advantages and drawbacks of the model system you have used as it relates to the disease you are investigating?**

For this study, we have studied β cell-specific pro-inflammatory cytokine induction as a proof of principle. By crossing our newly generated *Tg(CETI-PIC3)* line to the established *Tg(ins:cre)* line, we generated ins-CETI-PIC3 embryos in which we were able to induce Ifng1, Tnfa and Il1b specifically in β cells. Additionally, since this is a Tet-on system, our new zebrafish model system allowed us to induce these pro-inflammatory cytokines in a titratable manner, depending on the dose of doxycycline used. This novel model system allowed us flexibility by enabling us to precisely modulate cytokine induction using doxycycline dosing and timing. The system can be used to study aspects of any inflammatory disease for which an appropriate tissue-specific Cre is available.

**What has surprised you the most while conducting your research?**

Although it is known that the immune system senses stimuli and foreign bodies fairly quickly, I still found it very striking just how quickly the immune system responded to islet damage. Within 3 h of inducing pro-inflammatory cytokines in β cells, we observed an increase in macrophages, immune cells that are very responsive to cytokine signaling, surveilling the islet.

**Describe what you think is the most significant challenge impacting your research at this time and how will this be addressed over the next 10 years?**

I believe that making our research findings translatable to human disease is a significant challenge in the field. Nearly 70% of human disease genes have a functionally similar homolog in the zebrafish, making this model system a robust vertebrate model in which to study conserved aspects of disease. Additionally, the conserved aspects of zebrafish innate immune responses relative to mammalian systems, together with the ease of genetic manipulation of the model system, make the zebrafish a powerful *in vivo* model for studying the effects of excessive cytokine induction. However, to make studies in zebrafish relevant to human disease, studies in mammalian models and on human tissues also have to be performed. For example, to ensure that research findings are robust and translatable, studies can also be performed on human islets from pancreatic donors.

**Figure:**
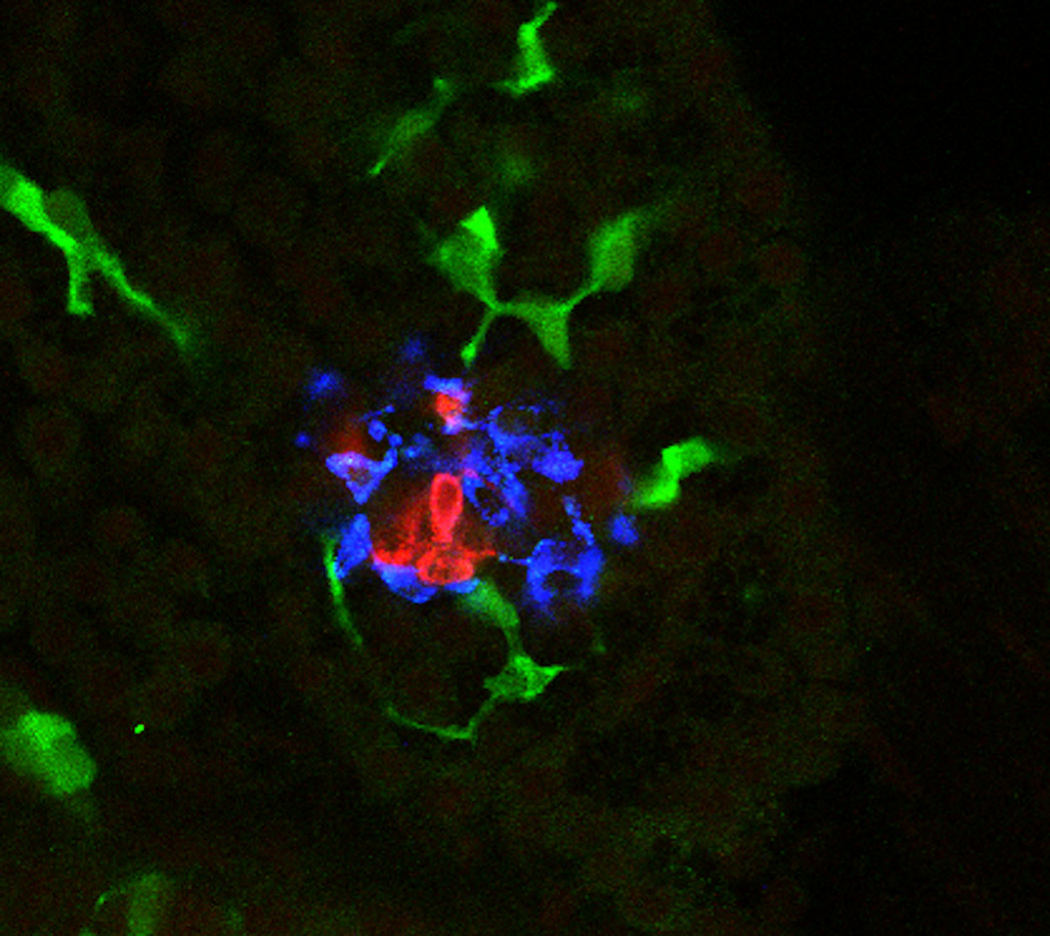
**Macrophages infiltrating into a damaged pancreatic islet.**

**What changes do you think could improve the professional lives of early-career scientists?**

I personally believe that having more funding opportunities/fellowships for early-career scientists to pursue pilot experiments will be very beneficial. This will allow for more creativity in science and will also cultivate more passion for research in early-career scientists.

**What's next for you?**

I will be heading back to finish the third and fourth year of medical school. After finishing my medical training, my career goal is to work in an academic medical center, where I hope to direct an extramurally funded research laboratory, along with seeing patients in the clinic.
